# Determining Medication Adherence in Patients Treated for Parkinson's Disease and Related Factors

**DOI:** 10.1155/padi/8889957

**Published:** 2025-09-26

**Authors:** Mahboobeh Besharatpour, Amir Kavousi, Mehri Salari, Koorosh Etemad

**Affiliations:** ^1^Department of Epidemiology, School of Public Health and Safety, Shahid Beheshti University of Medical Sciences, Tehran, Iran; ^2^Functional Neurosurgery Research Center, Shohada Tajrish Neurosurgical Center of Excellence, Shahid Beheshti University of Medical Sciences, Tehran, Iran

**Keywords:** drug-related side effects and adverse reactions, medication adherence, motor activity, neurologic examination, Parkinson's disease

## Abstract

Parkinson's disease (PD) is the second most common neurological disease. This study explores the determinant factors influencing medication adherence and disease stage among PD patients. This study was conducted on 161 PD patients at the Neurology Clinic. The eight-item Morisky Medication Adherence Scale (MMAS-8) and the International Parkinson and Movement Disorder Society Unified Parkinson's Disease Rating Scale (MDS-UPDRS) were used. Medication adherence and disease stage were analyzed using the chi-square test to measure the association of qualitative variables, and the Kruskal–Wallis test to test the association of quantitative data. Ordinal logistic regression was used to relate study variables with study outcomes. Medication adherence has a significant association with PD duration, number of times of taking PD drugs daily, comorbidity, total number of medications used, side effects, and history of medication discontinuation. The mean score of MDS-UPDRS subscales significantly differs. Medication adherence levels are suboptimal among PD patients, with significant correlations between medication adherence and disease stage, motor symptoms, and motor side effect. These insights underscore the critical need for targeted interventions to improve medication adherence and mitigate disease burden in PD patients.

**Summary**

• This study helps to improve treatment visits (long-acting medication) or care strategies for doctors, nurses, and patients' companions in managing the medication adherence of patients with Parkinson's disease.
• The information and influencing variables in the level of medication adherence of Parkinson's disease patients have been reviewed, and the results of their impact will help future research.
• Future research should look for a complete review of the types of specific medications for Parkinson's disease patients, as well as the use of evaluation tools, checking the level of medication adherence, and improving patients' quality of life.

## 1. Introduction

Parkinson's disease (PD) is the second most common neurodegenerative disorder, characterized by both motor and nonmotor symptoms. PD is the second most common neurological disease in the world after Alzheimer's disease, with an overall prevalence of 1.8 per 100 populations in people 65 years and older [1–3]. While less prevalent in younger people, who make up 5 to 10% of patients, PD is an age-related disease with an average onset of 57 years [4]. In normal people, with age, the amount of dopamine and the number of its receptors inside the basal ganglia decrease permanently, and apparently, this acceleration in reduction causes the incidence of PD [5]. According to research, the genetic factor plays a role in the involvement of Parkinson's patients in about 5%–15%. [6, 7]. In most PD patients, this disease is caused by genetic defects plus environmental factors [8]. At least 1–1.5 million people in the United States (US) live with PD, which is expected to double by 2030. In the US, the prevalence of PD is 128 cases per 100,000 people aged 50–54 years, which increases to 958 cases per 100,000 people aged 75–79 years [9]. It is also estimated that 5 million people worldwide have PD [3]. The total cost of PD in the US was estimated to be $23 billion in $2005 and $40 billion in 2040, including direct and indirect costs [10].

The World Health Organization (WHO) defines medication adherence as the degree to which the patient's behavior conforms to the recommendations agreed upon with a healthcare provider when taking medication.

Adherence to medications is the process by which patients take their medication as prescribed, further divided into three quantifiable phases: “Initiation,” “Implementation,” and “Discontinuation.”

• Initiation occurs when the patient takes the first dose of a prescribed medication.
• Discontinuation occurs when the patient stops taking the prescribed medication, for whatever reason(s).
• Implementation is the extent to which a patient's actual dosing corresponds to the prescribed dosing regimen, from initiation until the last dose.
• Persistence is the length of time between initiation and the last dose, which immediately precedes discontinuation [11].

One of the most important problems in the treatment cycle is the patient's non-cooperation in taking their medications correctly and completely, which is referred to as “suboptimal medication adherence” [12]. In the US, 33%–69% of hospitalizations are due to suboptimal adherence to medication, which leads to a cost of about 100 million dollars per year [13]. PD affects mostly people over 60 years old, and as the disease progresses, the medication dose reaches three or more steps during the day.

Several studies have reported suboptimal adherence in PD, which can lead to reduced efficiency, increased symptoms, early adjustments of the treatment protocol, and reduced quality of life [14–16]. Iran's population is rapidly aging, and since more than 6% of the country's population is over 60 years old, PD cases are estimated to increase to 10.5% by 2025 [17]. Therefore, following this increase in Parkinson's patients in our country, identifying their level of medication adherence can help create programs to reduce disease recurrence and control the disease in the early stages of the disease, identify complications resulting from discontinuation of treatment, and significantly reduce treatment costs for the individual and the health system. From the perspective of people's experiences (patients, families, doctors, and nurses), factors affecting medication adherence in PD have rarely been considered by researchers, we conducted the present study with the aim of determining clinical and nonclinical factors associated with medication adherence in patients treated at a major referral center for PD in Iran.

## 2. Methods

### 2.1. Study Design and Sample

A cross-sectional study was conducted with a convenience sample of 161 PD patients referred to the neurology clinic between 2022 and 2023. Inclusion criteria for this study included patients of any age and gender who had received a definitive diagnosis of PD according to the Movement Disorders Society (MDS) clinical diagnostic criteria for PD [18] and had been undergoing prescription anti-Parkinsonian medication for at least four weeks before enrollment. The exclusion criteria were patients who had been on medication for less than 4 weeks, or who did not consent to the study, or, if they had a chronic comorbidity, the comorbid conditions were other than cancer, diabetes, respiratory disease, or cardiovascular disease, or who were unable to respond due to the severity of the disease and old age and had no caregiver. Also, patients who had undergone surgery for PD.

Therefore, patients with other chronic comorbidities, including high blood pressure, chronic kidney disease, autoimmune disorders (rheumatologic disease), and mental disorders (depression), were excluded from the study.

The Movement Disorder Society Unified Parkinson's Disease Rating Scale (MDS-UPDRS) was reviewed and completed in person by the hospital neurologist for the patients before the start of the study and at the time of the start of the study; due to the impossibility of patients from different parts of the country to return to the hospital, the MMAS-8 questionnaire was completed by obtaining informed consent over the phone from the patient or his caregiver at the same time as the study began.

### 2.2. Clinical Outcome Assessments (COAs)

Patient-reported outcome (PRO) and its assessment are done through MMAS-8, and clinician-reported outcome (ClinRO) and its assessment are done through MDS-UPDRS [19].

#### 2.2.1. The 8-Item Morisky Medical Adherence Scale (MMAS-8)

This MMAS-8, designed by Morisky and his colleagues in 1980, is the most used COA to measure medication adherence PD, and it was suggested in 2023 by the MDS-COA program [18]. This self-reported COA evaluates the patients' behavior when taking medications. The first seven questions have two-way answers, except for Question 5, which has an inverse answer, and Question 8 contains a 5-point Likert COA answer. Higher answers indicate better adherence [20]. For Question 8, the score (0–4) should be standardized by dividing the result by 4 to calculate a total score [21]. The range of overall scores of this questionnaire is between 0 and 8. A score of eight indicates high adherence, a score of 6 and seven indicates medium adherence, and a score less than six indicates low adherence [22, 23]. The Persian translated version of this questionnaire has been used in internal studies with confirmed validity and reliability [24].

Permission to use the MMAS-8 questionnaire is provided by Dr. Morisky in the supporting information (available here).

#### 2.2.2. The International Parkinson and MDS-UPDRS

The MDS-UPDRS is the most used COA to assess PD symptoms [25]. It comprises nonmotor aspects (13 questions, with a score of 0–52), motor aspects (13 questions, with a score of 0–52), motor examination (18 questions, with a score of 0–132), and motor complications (6 questions, with a score of 0–24), which measure the severity of motor and nonmotor symptoms [19].

#### 2.2.3. Hoehn and Yahr (HY)

The PD disease stage is rated based on the disability scale of HY [26], which provides the overall estimate of the clinical aspects of PD, a combination of functional deficits (disability) and objective symptoms (impairment) [19]. This COA includes 0 (no signs of illness) to 5 (complete disability and use of a wheelchair) and includes 0, 1, 1.5, 2, 2.5, 3, 4, and 5.

### 2.3. Ethical Considerations

This article results from the master's degree thesis in the Faculty of Health and Safety of Shahid Beheshti University of Medical Sciences. The code of ethics of the thesis has been registered at Shahid Beheshti University of Medical Sciences under number IR.SBMU.PHNS.REC. 054.1401.

### 2.4. Data Collection

Based on the systematic review article [27], with a prevalence of *p*=0.727, a confidence level of 95%, *d*=0.1p, and considering a 10% sample dropout, the required sample size is 161 Parkinson's patients for the study.

Demographic information includes age, sex, marital status, education, employment, and social support status. Clinical information includes family history of PD, motor and nonmotor aspects, motor examination, motor complications, total number of drugs used, number of anti-Parkinsonian drugs used, number of times of taking Parkinson's drug daily, comorbidity, side effect, history of drug discontinuation due to side effects, and years of PD duration. The outcomes or dependent variables of the study include medication adherence and the PD stage, which are measured by the following tools.

### 2.5. Data Analysis

Demographic and clinical information obtained from the study were summarized and described using descriptive statistics methods in the form of mean, standard deviation, frequency, and percentage. The chi-square and Fisher's exact tests were used to assess the association between categorical variables and study outcomes. Also, to check the normality of the distribution of continuous variables, the Kolmogorov–Smirnov test was used; if it was normal, the analysis of variance was used, and if the assumption of normality was not established, the nonparametric Kruskal–Wallis test was used.

Linearity between continuous independent variables was checked by Spearman and Pearson correlation tests.

Assumptions of the proportional odds in ordinal logistic regression were confirmed in both outcomes: medical adherence level (low, medium, and high) and disease stage (unilateral, bilateral, bilateral with disability). Since the study outcomes are of an ordinal type, multiple ordinal logistic regression was used to evaluate the effect of factors with the categories of the medical adherence or disease stage. The significance level of all statistical tests was considered 0.05, and data analysis was performed using SPSS 26 and STATA17 software.

## 3. Results

Most of the 161 patients investigated, aged between 28 and 83 years old, were men (67.1%), married (88.8%), had nonacademic education (76.4%), and were retired (52.2%) (Table 1).

Most patients presented medium adherence to medication (47.8%).

In our study, all clinical variables showed a significant association with medication adherence.

Among the clinical variables, all clinical variables show a significant association. Among the clinical variables, the disease duration, number of types of Parkinson's medication, number of times of taking Parkinson's drug daily, the total number of drugs used, the history of drug discontinuation due to side effects, and side effect show a significant difference between the medication adherence categories.

Also, the average disease duration in the category of low medication adherence is 8.67 ± 4.24 years, which has the highest average disease duration compared to the medium and high medication adherence levels. The average number of drugs taken by patients in the low adherence category is 2.83 ± 1.73 and in the high adherence category is 4.44 ± 1.96, which shows that the more number of drugs a person takes is higher, the higher the medication adherence of those patients. Also, a history of drug discontinuation due to side effects was observed in 122 patients, of which 67 patients were in the medium adherence group and 43 patients were in the high adherence category.

Patients who had drug side effects, including movement disorders, mostly (33.8%) had moderate medication adherence, because these side effects reduced the patients' motivation to take their medications, which did not have high medication adherence.

Table 2 shows the results obtained from univariate ordinal logistic regression in order to investigate the association between medication adherence and disease stage with demographic and clinical variables.

The results show that there is a significant association between the clinical variables of PD duration, the number of PD medications per day, the total number of medications taken, the history of drug discontinuation due to side effects, and side effects with medication adherence categories, and between the variables of Parkinson's disease duration, side effects, and all subscales of nonmotor aspects, motor aspects, motor examination, and motor complications with disease stage categories.

The association between subscales with medication adherence is not statistically significant. Also, among nonclinical variables, there is a significant association between education and disease stage.

Table 3 shows the multivariate ordinal regression analyses of medication adherence categories according to demographic and clinical variables of Parkinson's disease. Therefore, the clinical variables of PD duration (years), the number of times of taking Parkinson's medication daily, comorbidity, total number of medications used, side effect, and history of drug discontinuation due to side effects have a significant association with medication adherence, among these variables, the number of times of taking Parkinson's medication daily(OR = 1.34), the total number of medications used (OR = 1.74), and the history of drug discontinuation due to side effects (OR = 24.28) had an odds ratio higher than 1 among medication adherence categories.

Table 4 shows the effective factors in the disease stage categories in terms of demographic and clinical variables and that clinical variables, motor examination (*p*=0.002) and motor complications (*p*=0.013), have a significant association with the disease stage. It should be noted that all significant variables had an odds ratio higher than 1.

Table 5 shows the results obtained from the association between the level of medication adherence and the stage of the disease by chi-square test, which shows that the association is significant (*p*=0.02).

In association to the stage of the disease, the highest medication adherence is related to patients with bilateral disease severity; in other words, patients who are in more severe stages of the disease and have movement disorders affecting both sides of the body have higher medication adherence to control their disease.

## 4. Discussion

The results showed that the highest percentage of Parkinson's patients in our study had moderate medication adherence, which is not in alignment with the research of Straka et al. [28] and Mandorf et al. [29]. The reason why our research findings are not nonalignment with those of Straka et al. and Mandorf et al. can be attributed to different measurement tools, different cutoff points, different medications used, and different sample sizes.

Other findings of the study showed that medication adherence in patients decreases with increasing duration of PD, comorbidities, and medication side effects.

This significant difference between medication adherence and the variable of PD duration can be attributed to the patient's reduced motivation in fighting PD.

Various medications can be prescribed to improve motor symptoms in patients with PD, these medications are often prescribed in combination in the middle to late stages of PD, and these medications have different dosages and administration schedules. Compliance with a single daily dose of a long-acting preparation of a dopamine agonist in the early stages of PD (e.g., ropinirole or pramipexole) may be simple, but in later stages of PD where symptom control requires the administration of more than one medication, the complexity of the medication regimen becomes can lead to a decrease in medication adherence in patients [30], Therefore, as the duration of the disease increases and PD progresses, the patient's adherence to medication decreases. In order to increase patient cooperation in this regard, there is a need to prescribe long-acting medications with fewer number of times used per day so that patients do not experience irregularity and sometimes helplessness in their treatment process and achieve better treatment results by taking medications regularly.

On the other hand, patients who have underlying diseases such as cardiovascular, respiratory diseases, cancer, and diabetes and are chronically and long-term involved in them, due to the increase in the number of daily medications and the complexity of the medication regimen, become tired and unmotivated from the process of self-control and treatment and do not take the medications regularly and as prescribed, and as a result, have lower medication adherence.

Also, during the treatment period for PD, the degenerative nature of the disease and the medications used in patients cause complications such as physical exhaustion and disability, impaired voluntary movements, nausea and vomiting, and the patient also suffers from psychiatric problems. Therefore, the occurrence of drug and treatment complications over time leads to a lack of continuous and regular adherence to medications, and the patient moves toward a decrease in medication adherence. Therefore, the role of medication adherence and its importance in better control of PD should be explained to patients and their caregivers in order to prevent complications during the treatment period as much as possible.

On the other hand, despite a history of drug discontinuation due to side effects, increasing the number of times Parkinson's medication is taken per day and the total number of medications taken, medication adherence in patients increases.

Because in the early stages of PD, the number of medications used increases slowly, and due to concomitant diseases, other medications are also limited in their use, so the patient is able to take a regular medication regimen and still has the physical strength to accept the medications, and on the other hand, they are psychologically motivated to control and treat themselves and thus have greater medication compliance; however, as shown in the research results, as the disease progresses from bilateral to bilateral with disability, this medication adherence decreases.

People who had a history of drug discontinuation during their treatment regretted stopping the drug due to the worsening of their physical condition and continued to follow their medication regimen, and as a result, they had higher medication adherence compared to other patients who did not have this experience of discontinuing medication.

In this regard, teaching psychological treatments, including medication adherence, to patients with PD increases the application of problem-solving strategies, increases self-confidence, increases self-discipline and control, and, as a result, can lead to increased medication adherence in patients [31].

Improving attitudes/beliefs about treatment and information exchange can increase medication adherence and in turn improve clinical outcomes [32].

Yet, there is no significant association between the level of medication adherence with the variables of age, job, nonmotor aspects, motor aspects, motor examination, motor complications, and the number of Parkinson's medications used. These results are alignment with the research results of Radojevic' et al. [33] and Aggarwal et al. [34].

Aggarwal et al.'s study showed that there was no significant difference between different age groups. In our study, there was no significant difference between different age groups, indicating that medication use was a priority across all ages and that age did not affect medication adherence.

Also, the employment status of Parkinson's patients did not differ significantly among the different adherence groups, as our study was not in developed, resource-rich regions of the world, where a significant association between medication adherence and employment status is observed. Therefore, living in urban areas did not show any significant differences in medication adherence patterns, suggesting that people living in urban areas have access to job resources for their illnesses, provided they are motivated to take medication. Therefore, due to the lack of significant differences in the occupational classes of the patients' families, no significant differences were observed between the different groups in medication adherence.

Other results of this study showed that there was no significant association between medication adherence levels and MDS-UPDRS subscales in Parkinson's patients, which is not alignment with the research of Grost et al. [35] and Malek et al. [30].

Most people scored worse on the MDS-UPDRS subscales as the disease duration and progression increased, which indicated patient deterioration and decreased medication adherence in all subscales; however, in our study, this worsening of the MDS-UPDRS subscales did not show a significant effect on reducing medication adherence in patients. One of the reasons for this was the presence of caregivers in the patient's family who monitored the patient's medication intake, especially during acute illness and worsening of the MDS-UPDRS subscales. Ultimately, it did not have any significant effect on medication adherence.

The findings also showed that there was a significant association between the stage of the disease and the variables of the motor examination and motor complications, because as the disease progresses, the patient's expectations about the effectiveness of the treatment may be unrealistic and lead to the patient's discontinuation of treatment.

In other words, irregular medication use in patients with early stages of PD does not have a significant impact on patient performance, but it will have a significant impact in patients with advanced PD, and as a result, it leads to a worsening of the “motor examination” variable in them, which this feeling of worsening after taking the medication leads to a decrease in medication adherence and it can hierarchically lead to an exacerbation of “motor complications” in the patient, and now, these patients with motor complications who are in the advanced stages of the disease, by changing their prescribed medication, reduce adherence to the medication, which can further lead to an exacerbation of motor complications in patients.

However, there is no statistically significant association between the stage of the disease and the variables of total number of medications taken, nonmotor aspects, motor aspects, gender, history of drug discontinuation due to side effects, education, comorbidities, duration of PD (years), number of Parkinson's drugs per day, and side effects in Parkinson's patients.

The results also showed that there is a significant association between medication adherence and disease stage, a finding consistent with the research of Mendorf et al. [36].

When Parkinson's disease progresses in patients and they enter from the unilateral stage of the disease (involvement of one side of the body) to the bilateral stage (bilateral involvement of the body), their medication adherence increases, but as Parkinson's continues and worsens to the bilateral stage of the disease with disability, medication adherence decreases because in the more severe stage of the disease, patients take multiple medications with higher doses, which causes a decrease in their medication adherence [37]. These increases usually lead to an exacerbation of side effects caused by anti-Parkinsonian drugs [38] and a wide range of side effects caused by high doses of levodopa, which ultimately reduce the patient's motivation and it reduces their medication adherence. In other words, the progressive course of PD requires different combinations and doses of anti-Parkinson's drugs, which has a negative impact on medication adherence and control of the patient's motor symptoms [39].

Since most people with PD (93%) live at home for an average of 10 years of their disease, therefore, the importance of the role of caregivers of patients in the intermediate to advanced stage is emphasized [40].

Thus, in the early stages of the disease, when medication regimens are simpler and daily doses are lower, there is a better prospect of adherence [30], but in more advanced stages, when medication regimens become more complex and daily doses are required, adherence decreases.

### 4.1. Limitations of the Study

In this study, there is a possibility of not reporting the actual amount of medication adherence and the possibility of information bias due to patients' self-reporting of medication adherence, as well as reducing the generalizability of the report results due to the collection of samples from a reference center, which causes selection bias. Failure to examine the dosages of the medication used and failure to examine more comorbidities can reduce medication adherence.

MMAS-8 has recall bias and acceptable response bias disadvantages. It also seems that due to the lack of information regarding the predictors of medication adherence, it is not able to comprehensively evaluate these reasons, which can lead to its weak association with objective clinical outcome measures and providing interventions to improve medication adherence and can only be considered good estimates of the medication taking behavior [41, 42].

## 5. Conclusion

Based on the research findings, it can be concluded that, in general, patients with Parkinson's do not have a favorable situation in terms of medication adherence, and its main determining factors include the PD duration, the number of times daily Parkinson's medication is taken, comorbidity, the total number of medications taken, the side effects of the medications, and the history of drug discontinuation due to their side effects.

Therefore, it is necessary to educate Parkinson's patients and their caregivers about the importance of medication adherence, as it improves medication use and patient treatment, reduces symptoms and duration of acute disease, reduces medication side effects, and reduces medical and healthcare resources and associated costs.

Further research is needed on other contributing factors, and more objective measurements are needed, if possible, including checking the number of times you take medication/prescriptions dispensed by physicians who have access to electronic prescription databases or by pharmacy-recorded data. Educating patients and their caregivers about appropriate care, collaboration between different healthcare providers, PD patients and their caregivers using a patient-centered approach, use of electronic adherence measurement devices with remote monitoring capabilities and medication reminder systems, use of short message service or alarm clocks, and smartphone apps were mentioned.

## Figures and Tables

**Table 1:** Examining the association between medication adherence with demographic and clinical variables.

Variable	Medication adherence categories				Univariate ordinal logistic
	Low level of adherence (41)	Medium level of adherence (77)	High level of adherence (43)	Total (161)	*p* value
Age					
< 60 years	26 (63.4)	33 (42.9)	23 (53.5)	82 (51)	0.10
> 60 years	15 (36.6)	44 (57.1)	20 (46.5)	79 (49)	
Sex					
Male	28 (68.3)	51 (66.2)	29 (67.4)	108 (67.1)	0.97
Female	13 (31.7)	26 (33.8)	14 (32.6)	53 (32.9)	
Education					
Academic	12 (29.3)	16 (20.8)	10 (23.3)	38 (23.6)	0.60
Nonacademic	29 (70.7)	61 (79.2)	33 (76.7)	123 (76.4)	
Married status					
Single and divorced	6 (14.6)	6 (7.8)	6 (14)	18 (11.2)	0.39^∗∗^
Married	35 (85.4)	71 (92.2)	37 (86)	143 (88.8)	
Job					
Yes	17 (41.5)	38 (49.4)	22 (51.2)	77 (47.8)	0.63
No	24 (58.5)	39 (50.6)	21 (48.8)	84 (52.2)	
Family history					
Yes	9 (22)	10 (13)	8 (18.6)	27 (16.8)	0.46
No	32 (78)	67 (87)	35 (81.4)	134 (83.2)	
Comorbidity					
No	31 (75.6)	61 (79.2)	34 (79.1)	126 (78.3)	0.85^∗∗^
Diabetes	5 (12.2)	11 (14.3)	5 (11.6)	21 (13)	
Cardiovascular	4 (9.8)	4 (5.2)	2 (4.7)	10 (6.2)	
Others	1 (2.4)	1 (1.3)	1 (4.7)	4 (2.5)	
PD duration (year)	8.67 ± 4.24	7.84 ± 5.77	6.16 ± 4.36	7.60 ± 5.11	0.014^∗^
More than 1 PD medication					
Yes	4 (9.8)	12 (15.6)	6 (14)	22 (13.7)	0.69
No	37 (90.2)	65 (84.4)	37 (86)	139 (86.3)	
Number of types of Parkinson's medication	1.49 ± 0.78	1.99 ± 0.91	1.53 ± 0.85	1.74 ± 0.89	0.001^∗^
Number of PD medications per day	3.98 ± 1.08	4.77 ± 1.74	5.37 ± 2.23	4.73 ± 1.82	0.004^∗^
Total medication	2.83 ± 1.73	3.12 ± 1.85	4.44 ± 1.96	3.40 ± 1.95	< 0.001^∗^
History of drug discontinuation due to side effects					
Yes	12 (29.3)	67 (87)	43 (100)	122 (75.8)	< 0.001
No	29 (70.7)	10 (13)	0 (0)	39 (24.2)	
Side effect					
No	3 (7.3)	17 (22.1)	17 (39.5)	37 (23)	0.03^∗∗^
Voluntary movement disorder	13 (31.7)	26 (33.8)	12 (27.9)	51 (31.7)	
Fatigue	3 (7.3)	3 (3.9)	1 (2.3)	7 (4.3)	
Both	16 (39)	27 (35.1)	11 (25.6)	54 (33.5)	
Others	6 (14.6)	4 (5.2)	2 (4.7)	12 (7.5)	

*Note:* Mean ± SD, number (%).

Abbreviation: PD, Parkinson's disease.

^∗^Kruskal–Wallis.

^∗∗^Fisher's exact test.

**Table 2:** The association between medication adherence and disease stage with demographic and clinical variables.

Variable	Medication adherence^1^		Disease stage^2^	
	Odds ratio (OR)^∗^	*p* value	Odds ratio (OR)^∗^	*p* value
Age				
< 60 years	1.29 (0.72–2.30)	0.38	0.98 (0.46–2.08)	0.96
> 60 years	Ref.		Ref.	
Sex				
Male	0.97 (0.52–1.80)	0.93	0.51 (0.23–1.13)	0.1
Female	Ref.		Ref.	
Education				
Academic	0.79 (0.39–1.59)	0.52	0.28 (0.10–0.82)	0.02
Nonacademic	Ref.		Ref.	
Married status				
Married	1.03 (0.39–2.70)	0.94	0.75 (0.23–2.42)	0.64
Single and divorced	Ref.		Ref.	
Job				
No	0.77 (0.43–1.37)	0.38	1.23 (0.57–2.63)	0.58
Yes	Ref.		Ref.	
Family history				
Yes	0.85 (0.38–1.89)	0.69	0.93 (0.33–2.62)	0.90
No	Ref.		Ref.	
Comorbidity				
Yes	0.87 (0.43–1.76)	0.70	2.03 (0.85–4.84)	0.11
No	Ref.		Ref.	
PD duration (year)	0.94 (0.88–0.99)	0.03	1.13 (1.05–1.22)	0.001
More than 1 PD medication				
Yes	1.25 (0.54–2.86)	0.59	1.13 (0.37–3.42)	0.82
No	Ref.		Ref.	
Number of types of Parkinson's medication	1.02 (0.74–1.41)	0.86	0.88 (0.57–1.35)	0.56
Number of PD medications per day	1.34 (1.13–1.59)	< 0.001	1.12 (0.92–1.36)	0.25
Total medication	1.35 (1.15–1.59)	< 0.001	1.16 (0.96–1.40)	0.11
History of drug discontinuation due to side effects				
No	28.98 (11.50–73.0)	< 0.001	0.56 (0.24–1.32)	0.19
Yes	Ref.		Ref.	
Side effect				
Yes	0.68 (0.55–0.86)	0.001	1.63 (1.20–2.21)	0.002
No	Ref.		Ref.	
Nonmotor aspects	1.02 (0.97–1.07)	0.46	1.14 (1.07–1.23)	< 0.001
Motor aspects	0.99 (0.96–1.02)	0.47	1.22 (1.14–1.30)	< 0.001
Motor examination	0.99 (0.97–1.01)	0.23	1.11 (1.07–1.15)	< 0.001
Motor complications	0.97 (0.92–1.03)	0.37	1.29 (1.18–1.41)	< 0.001

^∗^Univariate ordinal logistic regression.

^1^Medication adherence (high, medium, and low = Ref).

^2^Disease stage (unilateral, bilateral, and bilateral with disability).

**Table 3:** Multiple ordinal logistic regression to association between medication adherence categories with demographic and clinical.

Variables	OR (%95 CI)	*p* value
Age		
> 60 years	1.01 (0.46–2.20)	0.976
< 60 years	Ref.	
Job		
No	0.62 (0.28–1.37)	0.238
Yes	Ref.	
PD duration (year)	0.92 (0.85–1.00)	0.054
Number of PD medications per day	1.34 (1.08–1.66)	0.008
Comorbidity		
Yes	0.32 (0.12–0.87)	0.026
No	Ref.	
Nonmotor aspects	0.97 (0.90–1.05)	0.513
Motor aspects	1.04 (0.98–1.11)	0.123
Motor examination	1.00 (0.97–1.03)	0.824
Motor complications	0.960 (0.883–1.044)	0.343
Total medication	1.74 (1.35–2.23)	< 0.001
Number of types of Parkinson's medication	0.70 (0.45–1.11)	0.136
Side effect		
Yes	0.63 (0.46–0.85)	0.003
No	Ref.	
History of drug discontinuation due to side effects		
Yes	24.28 (8.65–68.16)	< 0.001
No	Ref.	

**Table 4:** Multiple ordinal logistic regression to association between disease stage and demographic and clinical information.

Variables	OR (%95 CI)	*p* value
Sex		
Male	0.31 (0.09–1.12)	0.076
Female	Ref.	
Education		
Academic	0.52 (0.014–1.92)	0.326
Nonacademic	Ref.	
PD duration (year)	1.04 (0.93–1.18)	0.436
Number of PD medications per day	0.97 (0.71–1.31)	0.827
Comorbidity		
Yes	2.14 (0.50–9.18)	0.303
No	Ref.	
Nonmotor aspects	0.97 (0.86–1.08)	0.581
Motor aspects	1.08 (0.99–1.19)	0.083
Motor examination	1.08 (1.03–1.14)	0.002
Motor complications	1.15 (1.03–1.29)	0.013
Total medication	1.30 (0.92–1.83)	0.127
Side effect		
Yes	1.17 (0.73–1.87)	0.506
No	Ref.	
History of drug discontinuation due to side effects		
No	1.70 (0.17–2.75)	0.607
Yes	Ref.	

**Table 5:** Association between medication adherence and disease stage.

Disease stage	Medication adherence		Chi-square
	Low and medium (118)	High (43)	*p* value
Unilateral	2 (28.6)	5 (71.4)	0.02
Bilateral	95 (74.8)	32 (25.2)	
Bilateral stage with disability	21 (77.8)	6 (22.2)	

## Data Availability

The data that support the findings of this study are available upon request from the corresponding author. The data are not publicly available due to privacy or ethical restrictions.

## Supporting Information

The MMAS-8 questionnaire is used to measure medication adherence in patients with Parkinson's disease, which is a valuable resource for assessing medication use behavior and addressing concerns about medication nonadherence. Therefore, a certificate of permission to use the MMAS-8 questionnaire was received from its author, Dr. Morisky.

Supporting Figure 1: MMAS-8 Questionnaire License.
