# Supplementary material for: Determining Medication Adherence in Patients Treated for Parkinson's Disease and Related Factors
Source: Parkinsons Dis. 2025 Sep 26;2025:8889957. doi: 10.1155/padi/8889957 (PMC12494472; doi:10.1155/padi/8889957)
Supplement: Supporting Information — Additional supporting information can be found online in the Supporting Information section. [file 8889957.f1.pdf]

1-Nov-2024

Certificate Number: 6374-9795-6358-0854-9198

To Whom It May Concern:

This is to inform you that Mrs. Mahboobeh Basharatpour, a graduate student in Epidemiology at Shahid Beheshti University of Medical Sciences (MSc) has my permission to use the MMAS-8 (Morisky Medication Adherence Scale 8 item U.S. Reg. No. TX-8-632-533) in this research study and for publication in all formats i.e. print and digital:

"Determining medication adherence in patients treated for Parkinson's disease and related factors in Shohadaye Tajrish Hospital."

The requirements to use this scale are to cite the following references in the document:

1. **Berlowitz DR, Foy CG, Kazis LE, Bolin L, Lonroy LB, Fitzpatrick P, et al. for the SPRINT Study Research Group. Impact of Intensive Blood Pressure Therapy on Patient-Reported Outcomes: Outcomes Results from the SPRINT Study. N Engl J Med 2017; 377:733-44.**

2. **Bress AP, Bellows BK, King J, Hess R Beddhu S, Zhang Z, et al, for the SPRINT Research Group and the SPRINT Economics and Health Related Quality of Life Subcommittee. Cost-Effectiveness of Intensive versus Standard Blood Pressure Control. N Engl J Med 2017; 377:745-55.**

Terms and conditions are found here:
https://adherence.cc/mmas-8

i. The following footnote is required in all articles, presentations, web postings, reports and submitted manuscripts, and on the first table or figure which present the MMAS-4 as well as in the Acknowledgment Section of manuscripts submitted for publication:

©MMAS 2006 www.adherence.cc

Donald E. Morisky, Sc.D., M.S.P.H., Sc.M. President

Philip Morisky, MBA
Chief Optimus
adherence.
www.adherence.cc
1-Nov-2024

student permission letter_Mahboobeh Basharatpour

Final Audit Report 2024-11-01

Created:	2024-11-01
By:	Philip Morisky (philm715@icloud.com)
Status:	Signed
Transaction ID:	CBJCHBCAABAAZdA9kIp1hiBMwLsuTLeufy7y3v5LCKT8

"student permission letter_Mahboobeh Basharatpour" History

Document created by Philip Morisky (philm715@icloud.com)
2024-11-01 - 2:47:06 PM GMT- IP address: 4.7.29.210

Document emailed to Philip Morisky (philip.morisky@adherence.cc) for signature
2024-11-01 - 2:47:10 PM GMT

Email viewed by Philip Morisky (philip.morisky@adherence.cc)
2024-11-01 - 2:47:19 PM GMT- IP address: 66.214.146.130

Document e-signed by Philip Morisky (philip.morisky@adherence.cc)
Signature Date: 2024-11-01 - 2:47:54 PM GMT - Time Source: server- IP address: 66.214.146.130

Agreement completed.
2024-11-01 - 2:47:54 PM GMT
